# Mechanical shock test simulation analysis of butterfly valves developed for the naval defense industry and evaluation of real test and production data

**DOI:** 10.1038/s41598-024-60302-4

**Published:** 2024-04-27

**Authors:** Erhan Ozkan

**Affiliations:** Dikkan R&D Center, Izmir, Turkey

**Keywords:** Mechanical shock test, Simulation, Computer aided design, Casting, Engineering, Materials science

## Abstract

The main purpose of this study is to investigate the mechanical shock behavior and develop the shock resistance of widely preferred butterfly valves for navy defense industries by handling the real test results with computer aided design and simulation programs. The 2D and 3D drawings were realized by using solid modeling and design programs. Mechanical analyzes to determine the mechanical strength of the specimens were carried out with the finite element analysis method by using structural simulation program. Mechanical shock test simulations were carried out by with shock response spectrum analysis. Solidification, filling-time–temperature analyzes, and simulation studies of inner stresses caused by micro and macro shrinkages were performed by using the casting simulation program. Comparisons of virtual tests simulated in computer environment with real tests were done in shock test setup. Products made of bronze were preferred due to the high corrosion resistance and the desire to be a useful research article that can respond to common applications in the defense industry. Virtual shock test simulation and real shock tests were performed according to the MIL STD 810 standard. The shock test results observations showed that by revising the design with a safety factor of 18% on the specimen, it was ensured that the product could pass the mechanical shock test even at an acceleration of 4000 m/s^2^. Then material become safe to use. With the use of a three-way feeder in the production of the reinforced design the difference in net weight from 19% has been reduced to 12%, while the production time has been improved by 22%.

## Introduction

The defense industry is responsible for the development and production of military technologies^1^. In this context, it carries out activities to protect against other states for the continuity of the sovereignty of a national state. Moreover, the defense industry consists of a commercial industry involved in research and development, engineering, production and service of military equipment, and facilities^2^. 92 percent of the defense industry employees are engineers. 30 percent of these are electrical-electronics engineers, 24 percent come from mechanical-materials engineering and 15 percent come from computer engineering^3–6^. These data once again brought up the importance of computer aided designs and simulations in the defense industry. Due to both the high cost and the high risk of the tests and international security measures, it has become inevitable to make these designs in a computer environment and to simulate the tests in a virtual environment^7–9^. Computer-aided designs come to the fore not only in the defense industry but also in the entire manufacturing industry^10^. In addition to the increasing needs of the defense industry, its integration and synergy with other branches of industry also play an active role. As it is known, all the technological developments are first realized in the defense industry and then spread to other sectors. Hence, it is possible to give examples ranging from the introduction of the internet into our lives to the inspiration of the equipment used in sports. Furthermore, the mechanical shock test was one of these applications^11–13^.

**Figure 1:**
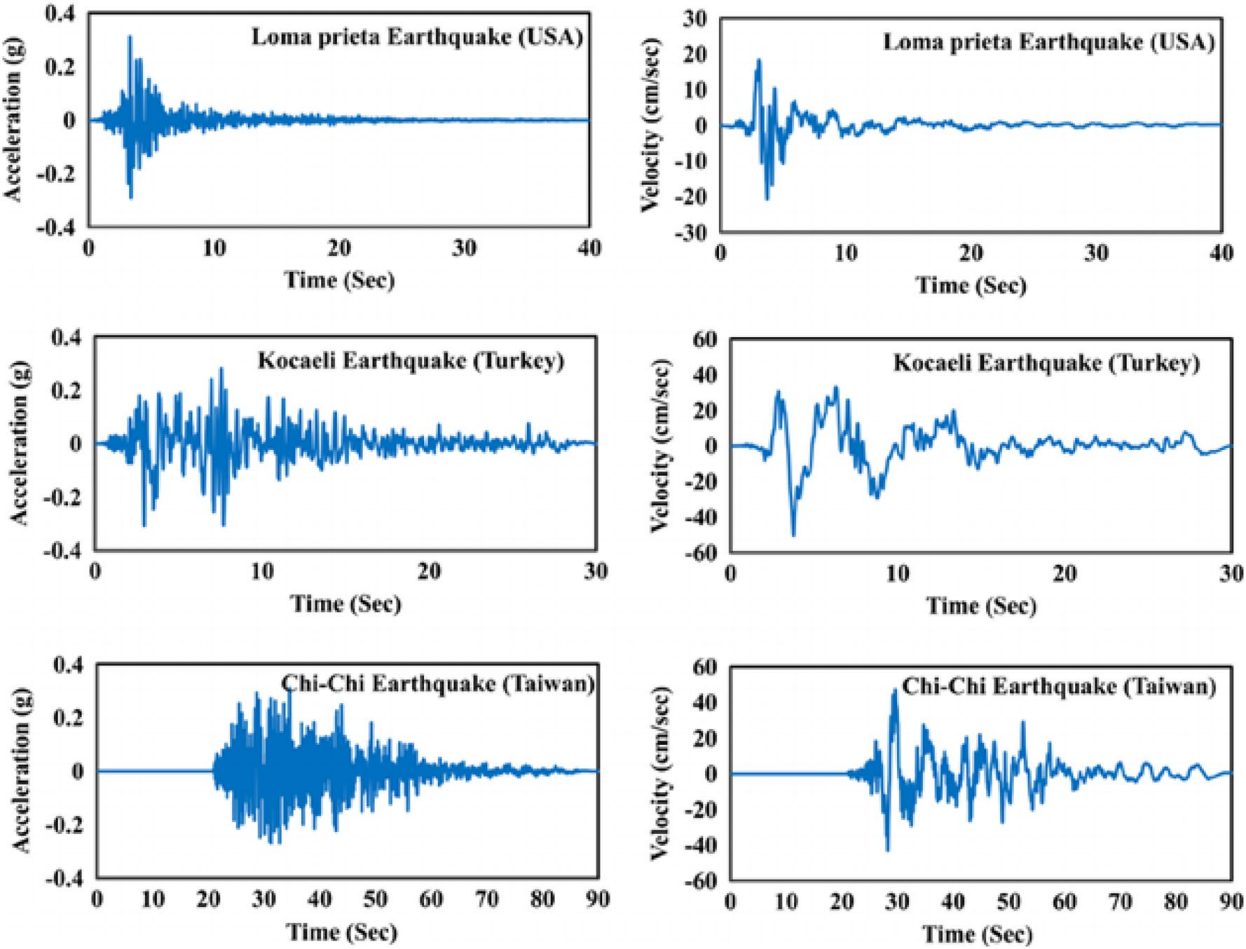
Time vs Acceleration-Velocity History of the Different Type of Seismic Actions.

Mechanical shock can be defined as a short-term, high-amplitude force effect applied to the material, causing sudden acceleration of the material^14^. Non-periodic stimuli such as impact, fall, explosion, cannon fire and earthquake can cause sudden velocity and acceleration changes on the material. Due to these effects, mechanical shock can disrupt the physical integrity of the materials as well as negatively affect their functionality. The magnitude of the possible physical and functional effect on materials may vary depending on the amplitude of the shock as well as the duration of the shock. In this context, the shock profile must be analyzed correctly^15–19^. In the real world, when the conditions that cause mechanical shock are examined, the time-dependent acceleration, velocity and displacement amplitude information is seen as in the example shared below as Fig. 1^20^.

Due to the difficulties encountered in the mathematical expression of the sample and other similar measurement results and the inability to create these measurement values ​​correctly in the laboratory environment, some shock signals corresponding to mechanical shock have been defined. The most used of these signals are half sine, saw tooth, square type shock signals. With the use of deterministic signals, system analyzes can be done in a shorter time and more easily, and it is also easier to compare signals with each other. Being able to compare different types of signals correctly, the available data must be processed equally. For this purpose, methods such as SRS (Shock Response Spectra), ESD (Energy Spectral Density), FS (Fourier Spectra), TDM (Time Domain Moments) and EM (Energy Methods) can be used^21–24^.

The resistance of defense industry products to environmental conditions is defined in the MIL STD 810 standard and it is aimed to determine the reactions of the products to the most difficult environmental conditions. Mechanical shock testing equipment and systems for naval applications must be consider detailed for the materials science together, hence the corrosion and mechanical resistance becomes important for these applications^25–27^.

Computer Aided Design (CAD) is the use of computer systems to assist in the discovery of a part or construction that is intended to be manufactured. More simply, the part that is intended to be manufactured is first created in a computer environment. Thanks to CAD, designers and engineers can realize their designs in electronic environments. CAD is a software program used to help create, modify, analyze, or optimize a design. CAD software is used to increase designer productivity and design quality, improve communication through documentation, and create a database for production. Different programs can be used for three-dimensional and two-dimensional designs. PLM NX, SolidWorks, Autodesk Maya, AutoCAD, CATIA are some of these programs. In this way, they can make changes on their designs much more quickly and print this electronic information on paper whenever they want^28^. Designers can assemble the parts they have completed and thus easily check whether the design they have made will work properly as a whole. Designers initially experience what they can produce with software by making two-dimensional designs. As they produce and learn, they create different visuals by applying image and graphic processing software better. Different programs can be used for two-dimensional and three-dimensional designs^29^. One of them, ANSYS analysis software, is used to analyze strength, toughness, elasticity, temperature distribution, electromagnetism, fluid flow and other properties as well as computer models of fluid dynamics, structural mechanics, electromagnetic, Multiphysics and systems simulation. Many examples such as the automotive industry, aviation and space, energy technologies, heating and cooling sector, ventilation industry, processing of coal, oil and natural gas, renewable energy sector can be counted as users of this simulation system^30–35^.

In this study, the contribution of the parameters to the shock test and their effects on the structural properties in the simulation-supported design of butterfly valves, which have not yet been encountered in the literature and will find widespread application in the defense industry, were explained in detail. Thus, an original study on the changes that occur due to changing parameters in synthesis studies carried out on the experimental setup has been introduced to the literature. Moreover, the computer aided designs, mechanical simulations supported by finite element methods, virtual and real tests of the butterfly valves developed for the naval defense industry applications were presented with details. According to the results obtained, the data on the prolongation of the service life of samples with different design and application parameter values were transferred in detail, and technological solution methods were proposed by disseminating the obtained results. In addition, the test conditions and production parameters with their interactions and changes with the mechanical shock behavior of the designs were presented to provide additional information to the readers.

## Material and method

### Design

**Figure 2:**
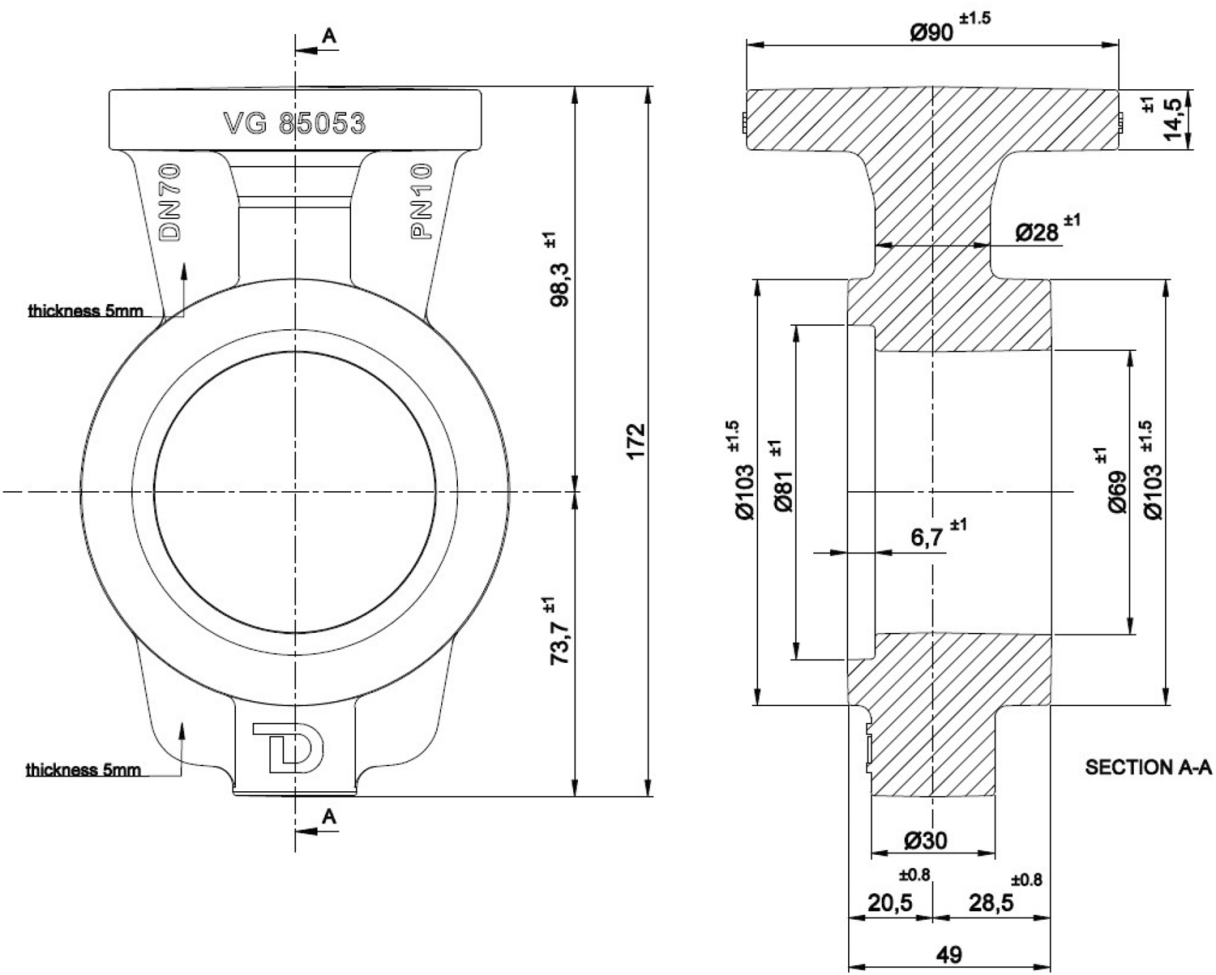
2D Technical Drawing of DN 70 PN 10 VG Butterfly Valve (Widely Used Design).

Bronze valves, which are widely used due to their resistance to atmospheric corrosion, were preferred as a test sample, and it was chosen as a butterfly valve designed to comply with the VG 85,011–3 connection and VG 85,053 face to face size standards. The designs of the specimen to be cast and tested were completed with the help of CAD. This phase provides the basis for generating data for the design simulation. Two-dimensional (2D) pictures of the valves to be tested were drawn in AutoCAD and shown in the Fig. 2.

**Figure 3:**
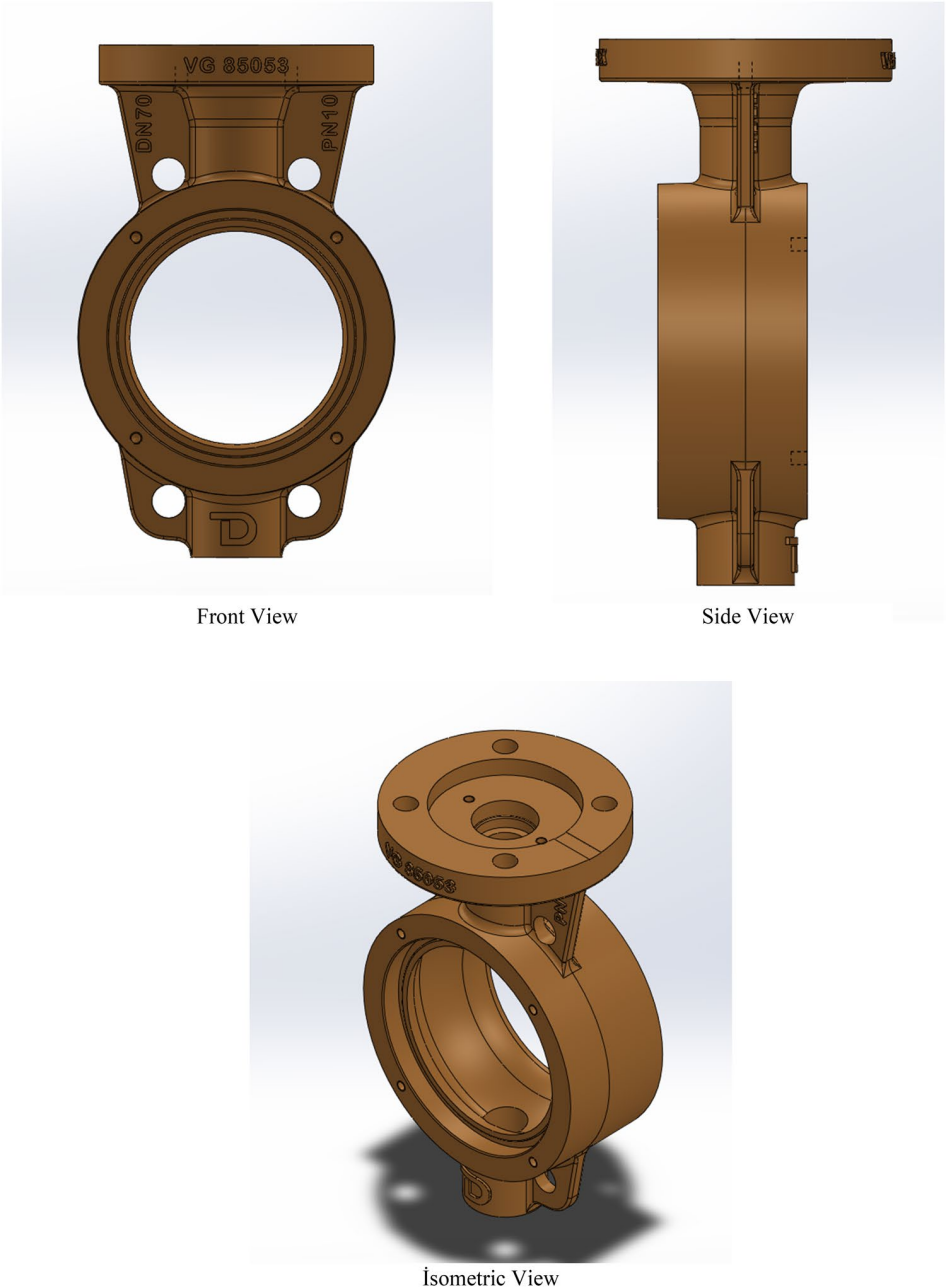
Views of the First Design to be Tested from Different Angles (Widely Used Design).

2D drawings provide the basis for the design, as well as giving the designer an idea for three-dimensional (3D) drawings. The 3D drawings of the samples to be tested were made with SolidWorks 3D solid modeling and design software. The Fig. 3 shows the front view, side view and isometric view of the test specimen.

### Simulation

**Figure 4:**
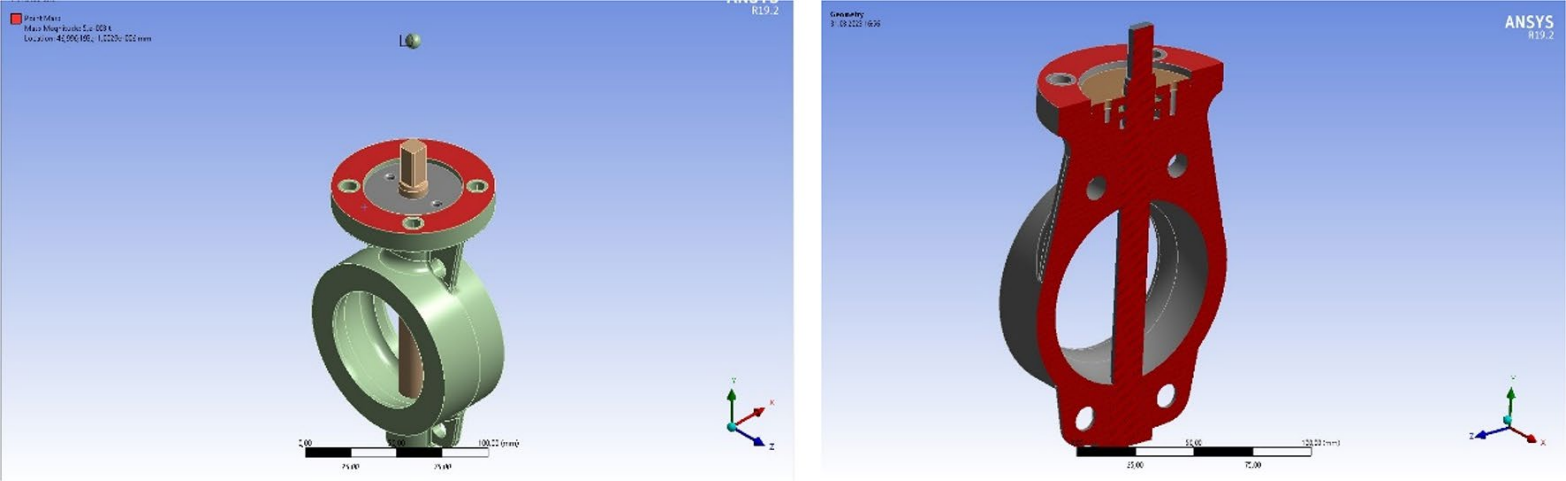
Ansys Geometry Analysis of the Specimen (Widely Used Design).

Simulation is one of the critical processes to determine the mechanical characterization of the material before the real test. Therefore, to determine the mechanical strength of the material and optimize the design according to the limit values, mechanical analyzes were carried out with finite element method (FEA) in the ANSYS simulation program. ANSYS geometry analysis was performed with the integration of 3D solid models prepared in SolidWorks. The point mass region that represented to test equipment connection and the section of the geometry were given in the Fig. 4.

**Figure 5:**
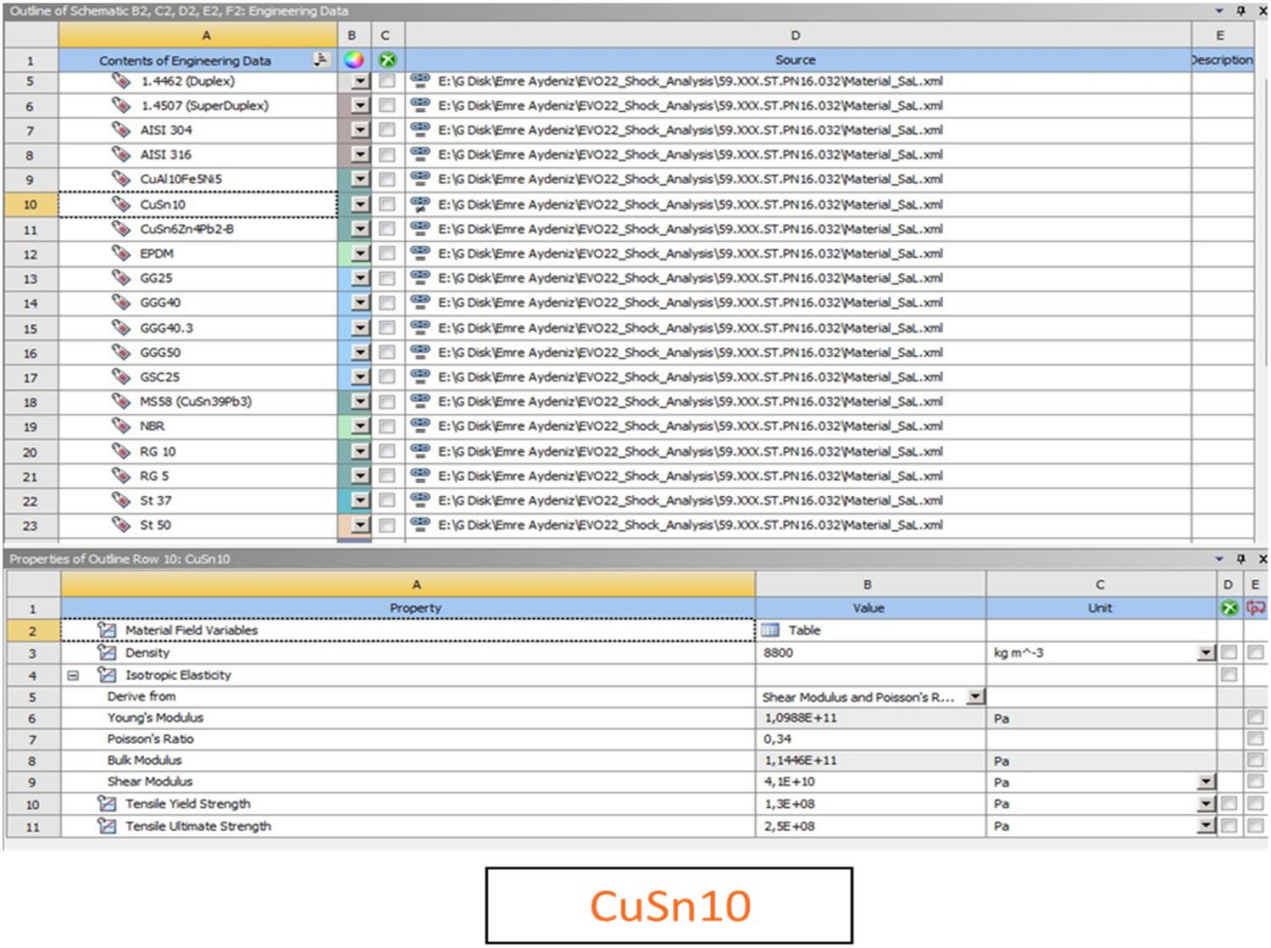
Material Selection Screen to be used for Analysis.

CuSn10 (C90700 / SAE 62, DIN 1075—2.1050, CC480K) material was chosen from the ANSYS library to ensure the high corrosion resistance and fulfill defense industry expectations. The Fig. 5 shows the details of the CuSn10 material selection information from the ANSYS library.

**Figure 6:**
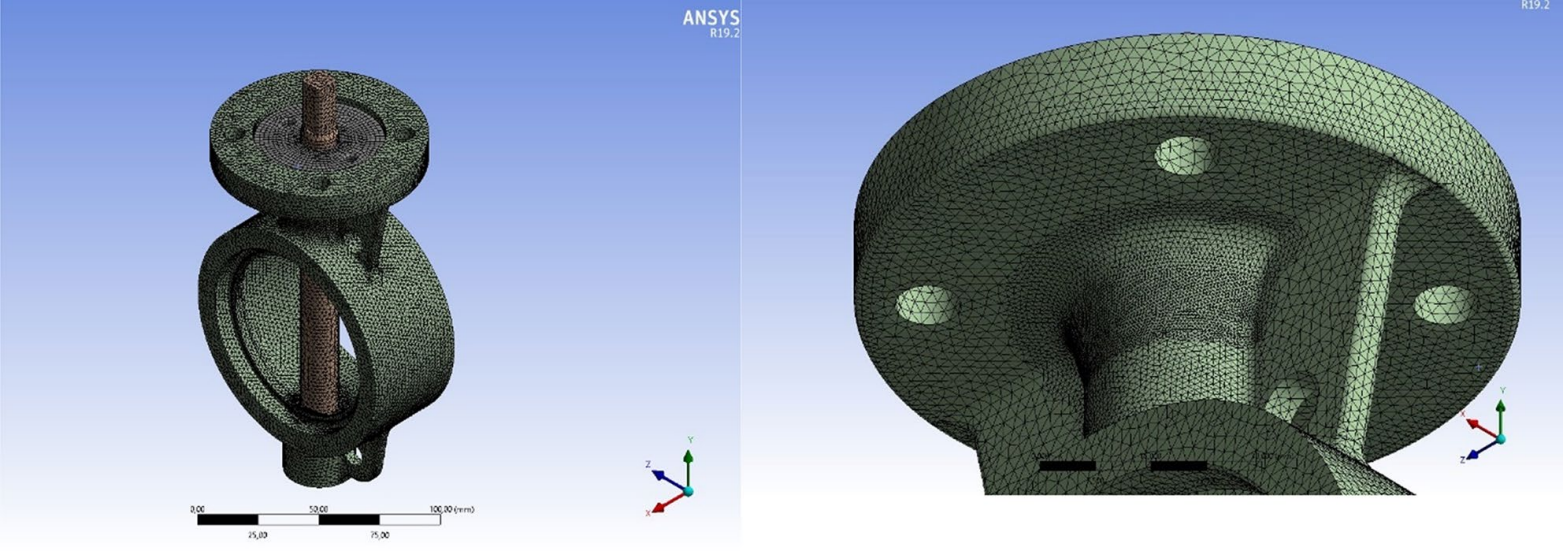
Mesh analysis of the specimen.

Mesh analysis was carried out to perform ANSYS mechanical and shock analysis module. In the finite element model, solid structures were modelled with SOLID 186 and SOLID 187 element types. SOLID 186 element is an element type that has three-dimensional quadratic shape functions and contains 20 nodes. SOLID 187 has the same three-dimensional quadratic shape function, but there are a total of 10 nodes. Each node of both element types has three degrees of freedom in the transaction direction: x, y, and z. The purpose of the mesh was to break a complex volume into small segments to be simulated. The mesh defined here consists of cells and points. It could have almost any shape in any size. It was used to solve Partial Differential Equations. The mesh analysis of the specimen is given in Fig. 6. The element quality and aspect ratio are quite important for checking mesh and element quality. Therefore, the aspect ratio was handled minimum 0.38 and maximum 10 respectively, where the tolerances were defined in MIL STD 810.

**Figure 7:**
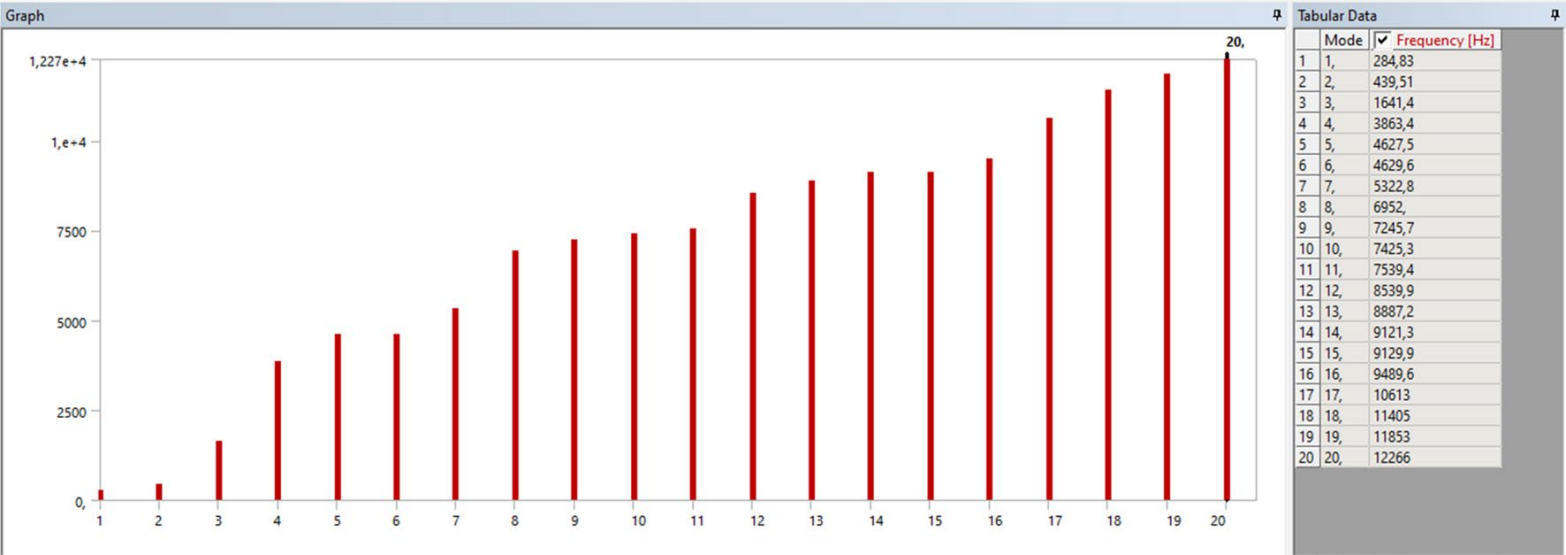
Shock test model selection.

A model was determined for the ANSYS shock test simulation and the basis of the tests was established. Test standards parameters were taken as a basis for model determination. Model details are given in the Fig. 7. DoF represents degree of freedom and has been chosen as 20 in accordance with the standards. Moreover, the natural frequency was set as 284 Hz because it is suitable for the frequency on which the real test would be performed, and it was close to reality.

**Figure 8:**
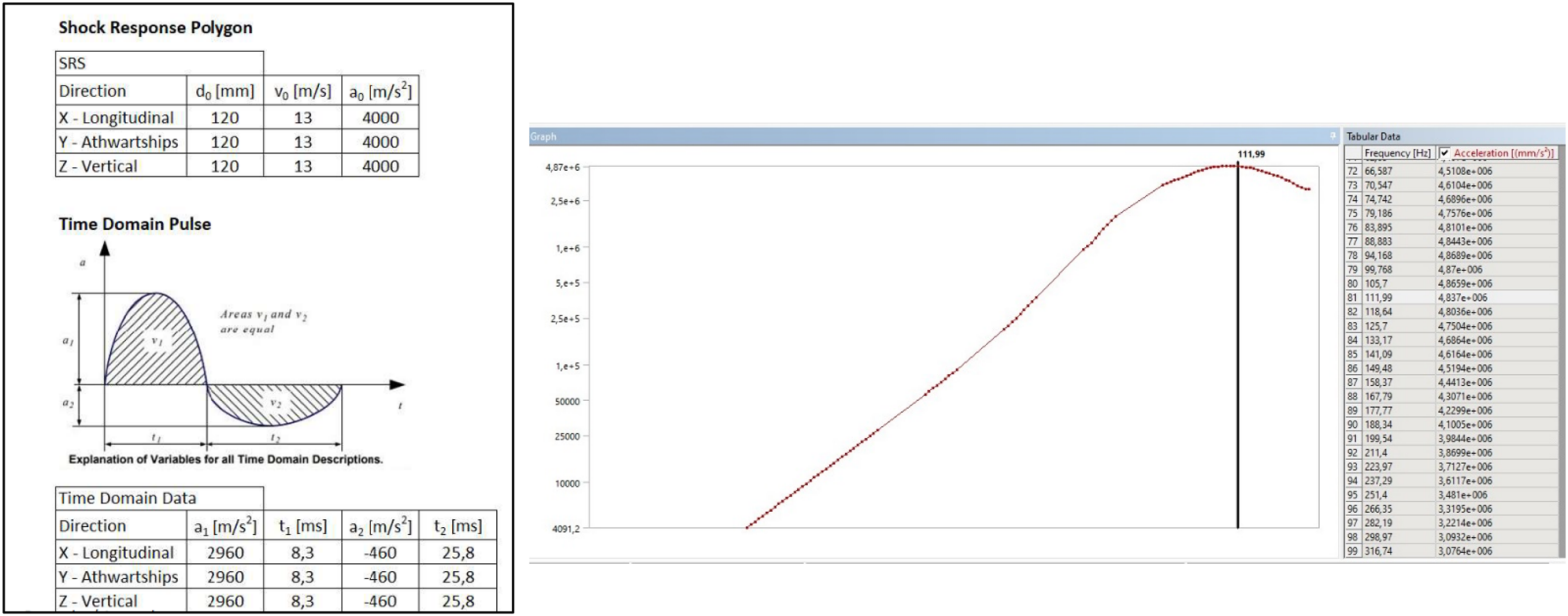
Shock response selection for the ANSYS simulation.

The Shock Response Spectrum (SRS) method was first used in military standards, then standardized with ISO 18,431–4 as a signal processing method. Here, in summary, time-dependent signal information was converted into frequency-dependent acceleration information. Due to the difficulties encountered in the mathematical expression of the measurement results and the inability to create these measurement values correctly in the laboratory environment, some shock signals corresponding to mechanical shock have been defined. The most used of these signals are half sine, saw tooth, square type shock signals. The shock test wave selection as half sine, which was selected for the analysis and would also be used in the actual test, is shown in the Fig. 8.

**Figure 9:**
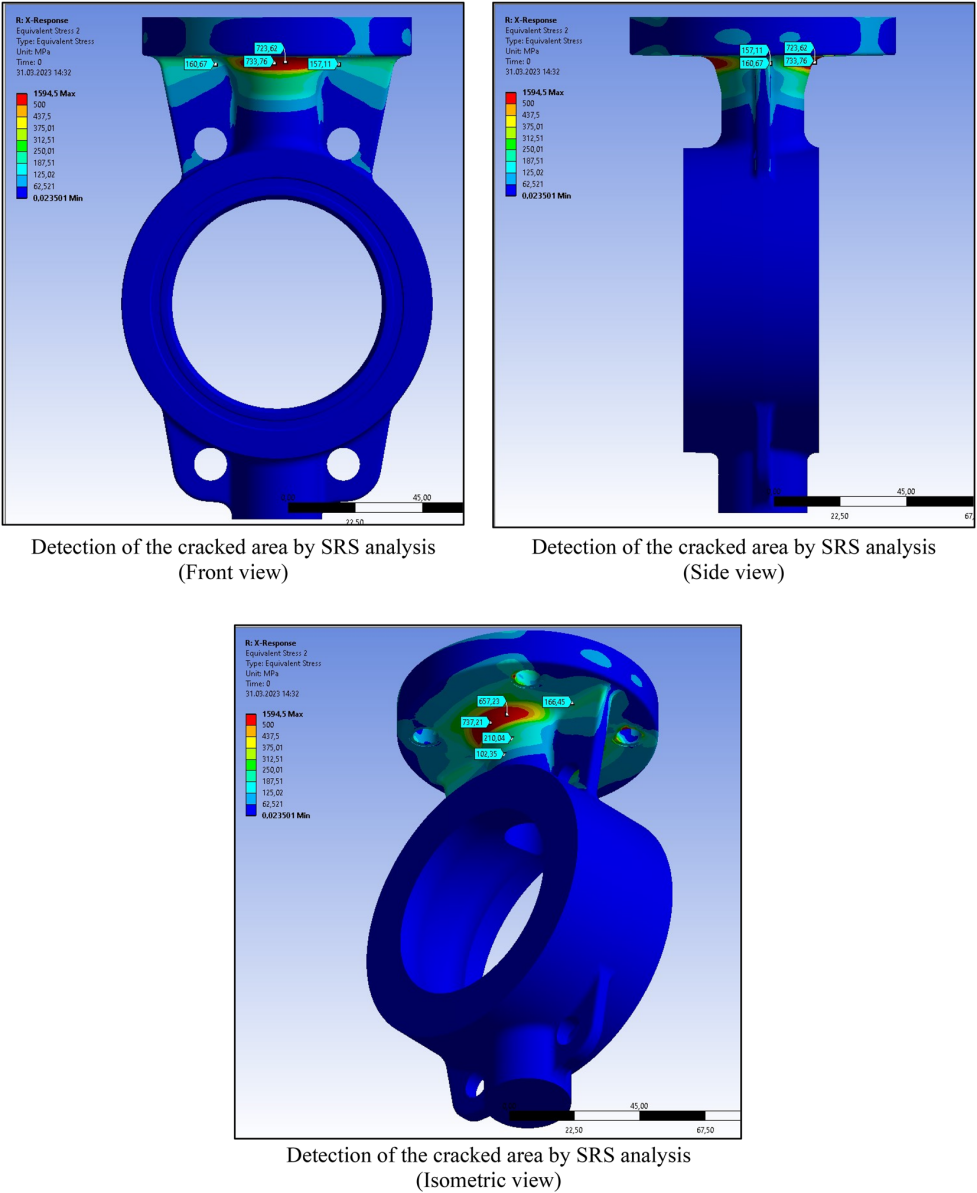
Ansys SRS (shock response spectrum) analysis for stress and deformation.

Shock analysis simulation was performed after all product, test and field parameters were defined to the system. Figure 9 shows the shock response spectrum for the different sides. The red lines and red areas represent the cracked region detected by ANSYS SRS analyses.

### Tests and characterization

**Figure 10:**
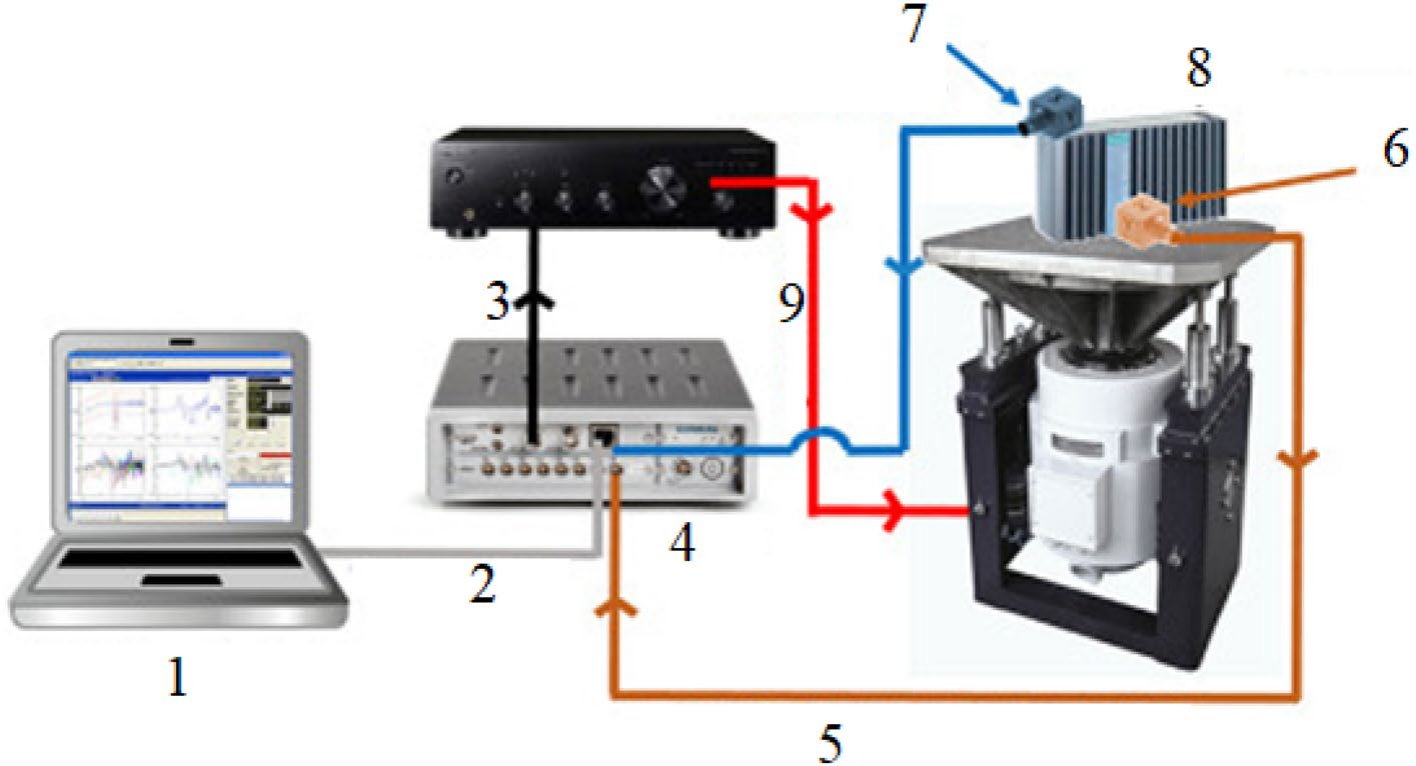
Shock test control diagram.

The samples were exposed to shock test to observe the ANSYS simulation data in real environment. The components of the shock test control diagram represented by numbers in the Fig. 10; (1) the PC control system, (2) ethernet to Scada, (3) Scada output to DC system, (4) Scada, (5) control accelerometer to input of Scada, (6) control accelerometer, (7) measurement accelerometer, (8) test specimen, and (9) DC system output to the seismic mass respectively.

**Figure 11:**
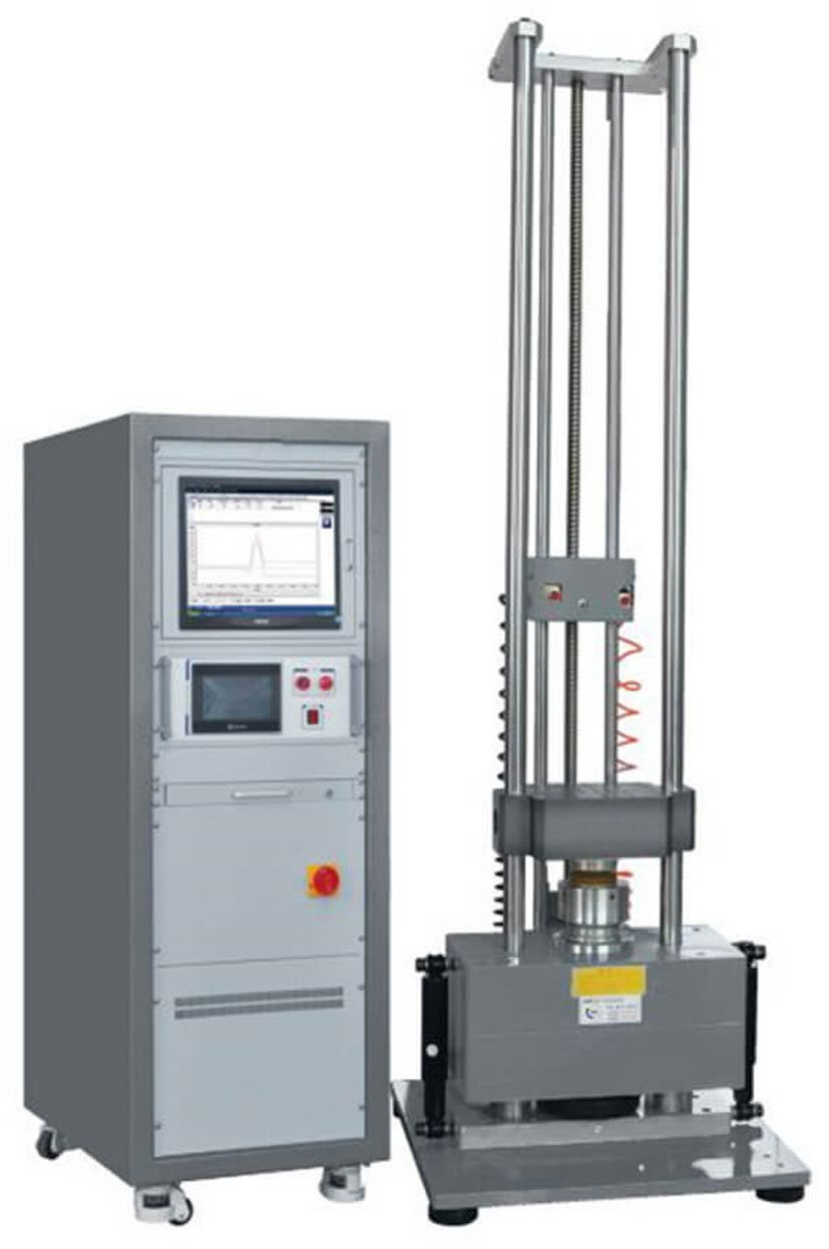
Mechanical shock test system.

A self-check must usually be performed by the software before the shock test is carried out to ensure that the entire system (controller, amplifiers, sensors, shaker, test objects, …) is functioning and assembled properly. Scada data acquisition system was used to convert analog signal from accelerometers to digital signal and send the converted signal to computer. It also allowed analog signal output to the amplifier to regenerate the shock wave at certain levels. A product's ability to withstand mechanical shocks was both a key design and a key selling point for certain military and consumer goods. Therefore, a dynamic mechanical analysis was a necessary component of quality control and product testing. Typically, a range of shock levels might be defined by idealized shock response spectra. Several navies have different design philosophies, which may consider location of equipment on board (i.e., shock zones) or other parameters. The shock test equipment in which these details were tested is shown in Fig. 11.

Products used on naval vessels must be able to withstand a variety of mechanical shocks, including everything from general turbulence to impacts from enemy regulations. These products must meet the requirements of MIL-S-901 standards. The shock test parameters that provide these standard requirements are given in Table 1^36^.

**Table 1:** Parameters employed during mechanical shock test (MIL-S-901).

Limit load	12 kg, (with test specimen)
Dimension of the Bench	L225 × W225 mm
Wave Type	Half Sinusoidal
Acceleration of the Shock Impact	160 ~ 16,000 m/s^2^
Duration	0.6 ~ 20 ms
Form of Impact	Free fall
Height of Drop	1600 mm
Working Temperature	25 ~ 40℃
Humidity at 25℃	< %80

Valves made of bronze, which is widely preferred in defense industry ships due to its high corrosion resistance, were used in the test samples. The chemical analysis, mechanical and physical properties of the material known as CuSn10 and defined as Standard Alloy Number (C90700 / SAE 62, DIN 1075—2.1050, CC480K) was given in Table 2^37^.

**Table 2:**
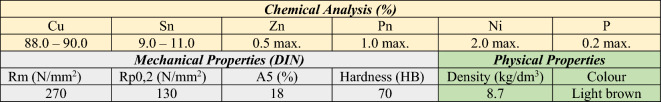
Chemical, mechanical, and physical properties of the shock test specimens (DIN 1075).

**Figure 12:**
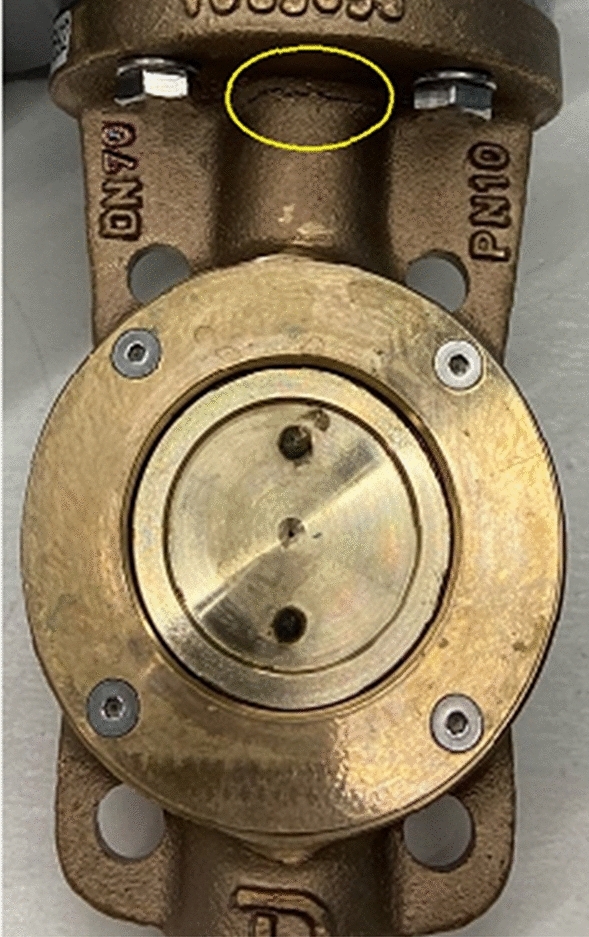
Crack after shock test (widely used design).

The image of the damaged sample after the shock test was completed is as shown in Fig. 12. The damage detected in the ANSYS simulation exactly matched the actual test. However, there was no corrosion-induced defect or wear on the specimen surfaces.

**Figure 13:**
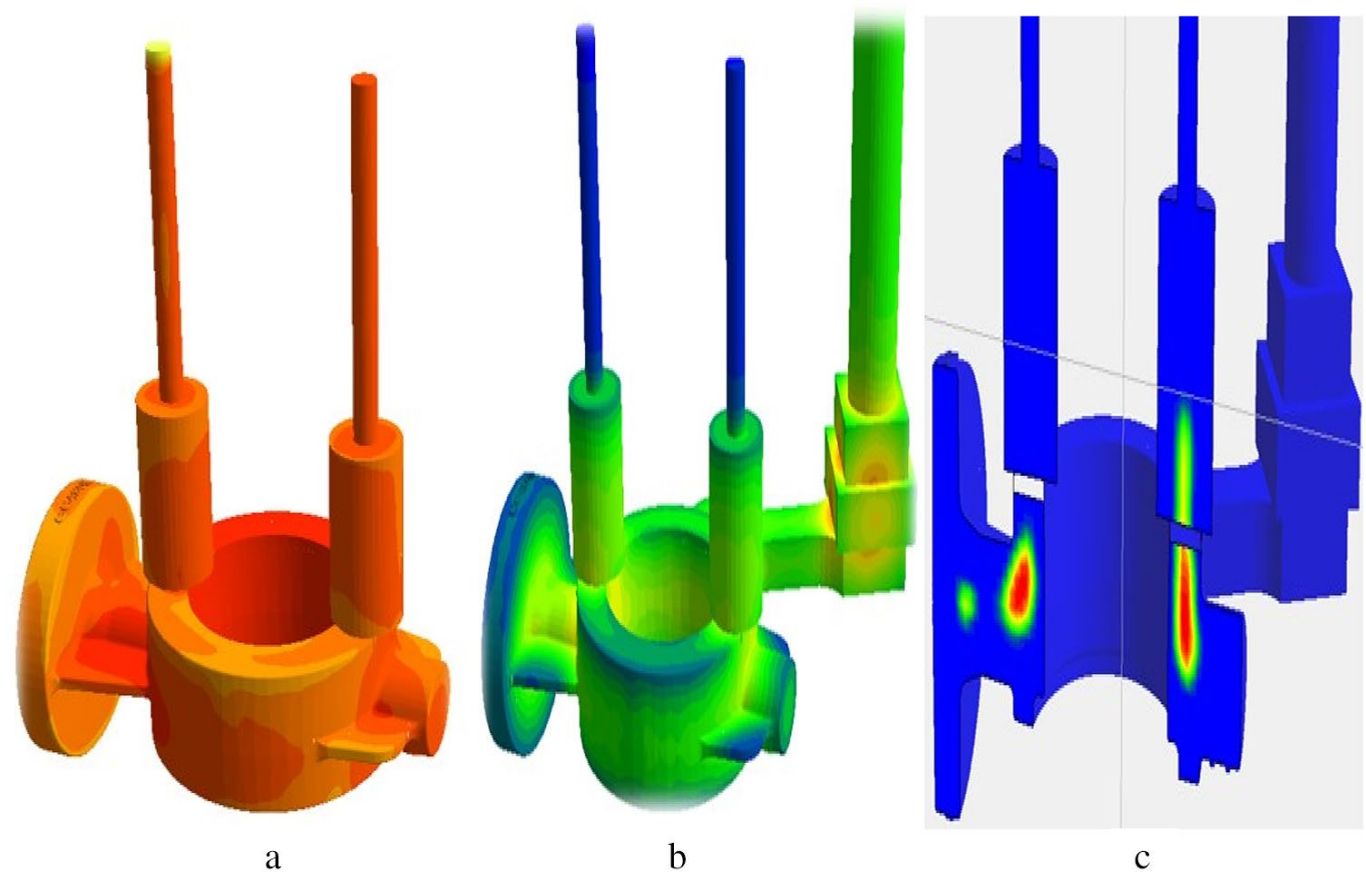
Casting simulation results; (**a**) Heating, (**b**) Solidification, and (**c**) Final product inner stress areas.

Following the negative result from the shock test, casting analysis was performed with the Anycasting simulation program to detect errors that might arise from casting in the damaged area. The design was carried out with double runner feeding in the Anycasting simulation program for CuSn10 material at 1150 °C as a casting temperature. According to the simulation data, the casting process took 19 s. Figure 13 shows the casting simulation heating, solidification, and inner stress analyses.

It has been determined that the stresses formed in the regions where micro and macro shrinkages were observed in the simulation cause errors and crack in the shock test from this region. This situation was clearly observed in the red regions detected in item c in the Fig. 13.

**Figure 14:**
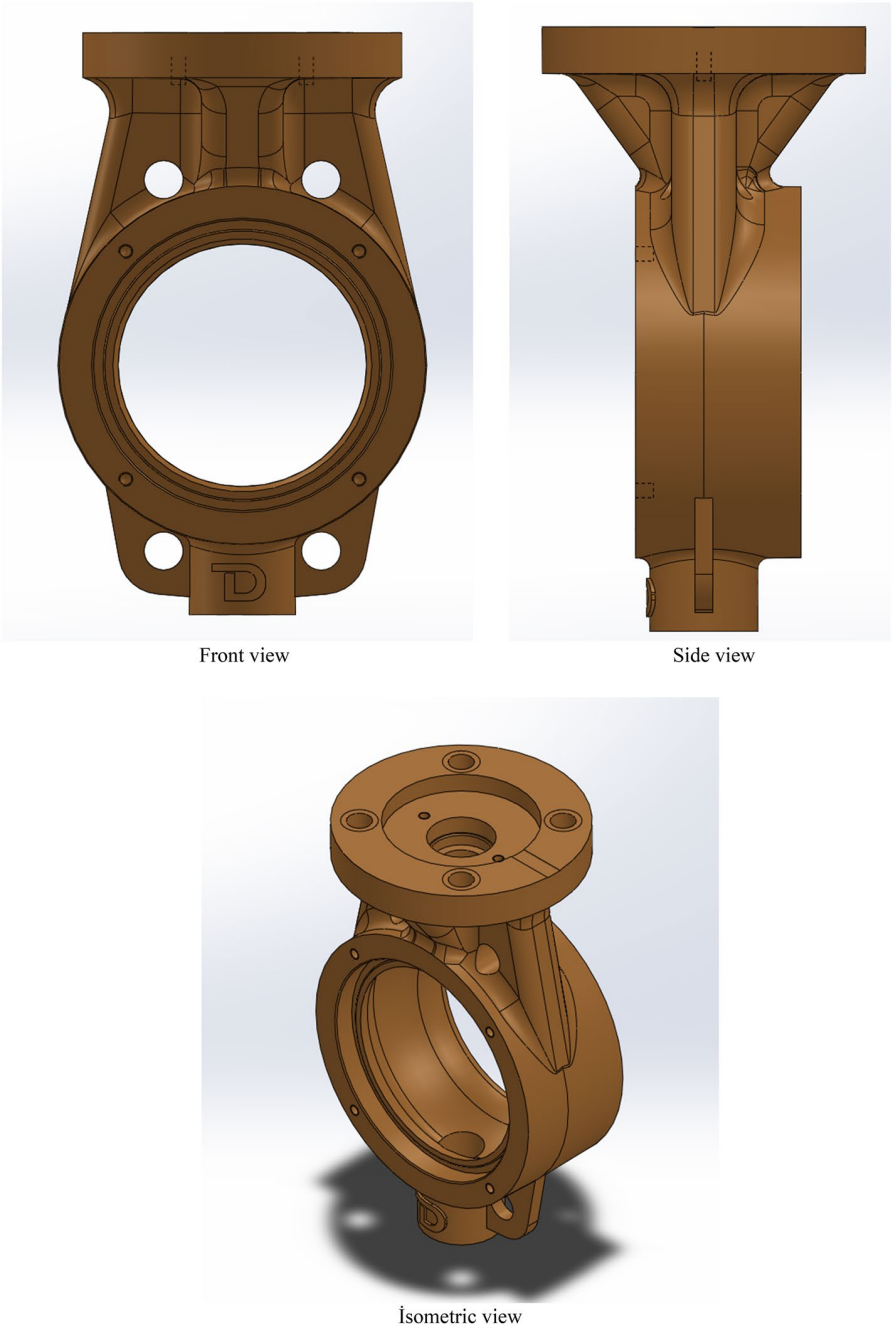
Revised design for the retest (own design).

The design was reconsidered to prevent this situation, and feeder reinforcements were made to the areas with shrinkage. Figure 14 shows the revised design of the samples that would be exposed to the mechanical shock test. This original design was developed by R&D center researcher. Even though there are similar designs in the industry, the originality in the production method distinguishes this design from its similar ones.

**Figure 15:**
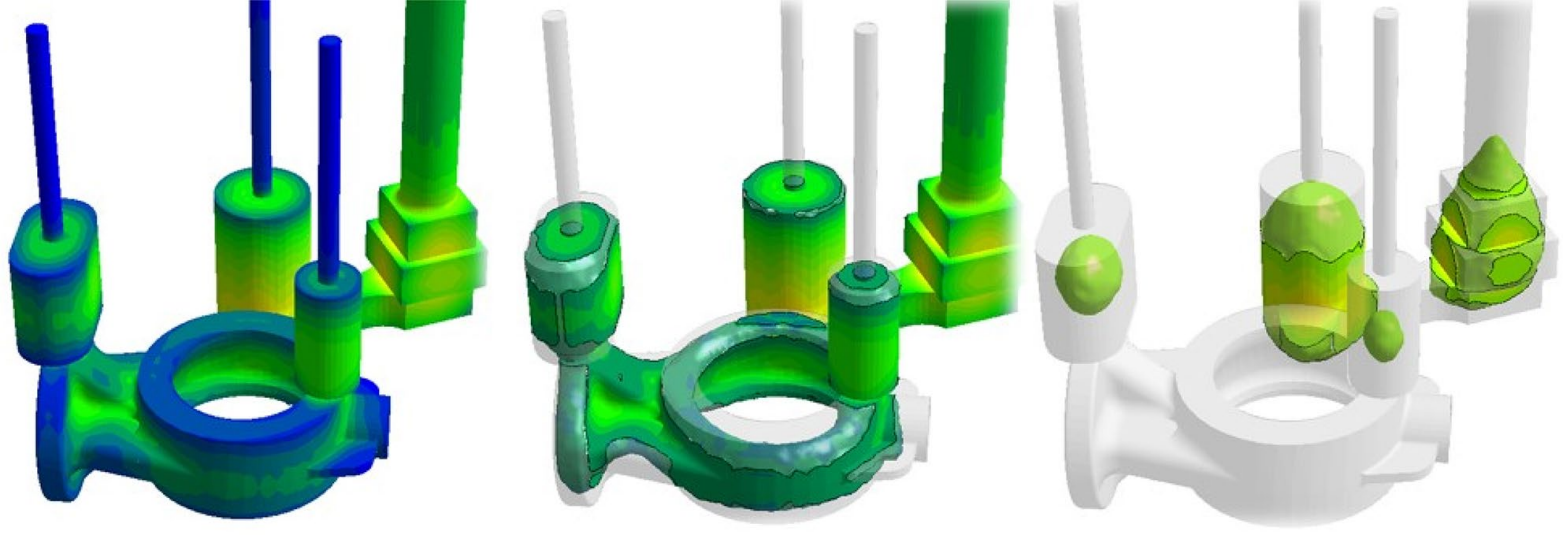
Solidification simulation of the new reinforced sample (own design for the process).

Casting simulation was performed to determine the effect of the change in design on the casting process, and it was observed whether the parts of the product with broken detected were improved or not. However, with the reinforcements in the design, the increasing thickness on the casting was optimized and a three-way feeder was simulated for solidification (Fig. 15).

**Figure 16:**
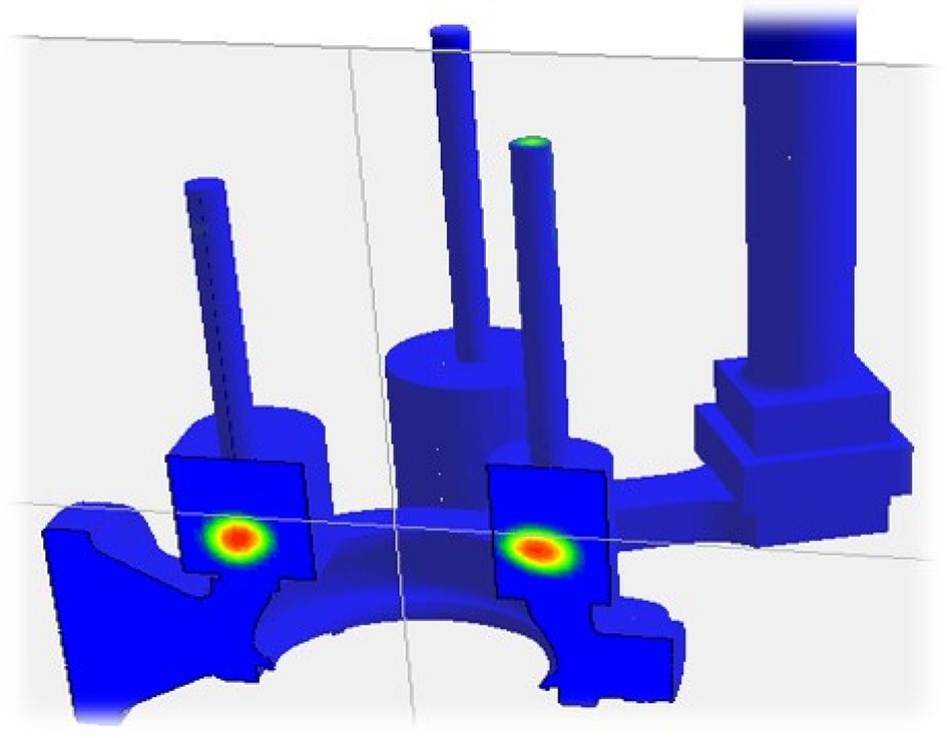
New (own) design casting simulation shrinkage, stress analyses.

As seen in the Fig. 16 no detection, shrinkage or inner stressed area were observed. All stresses remained in the runners and feeders that would be separated from the base material and no red area reflected on the sample was observed.

**Figure 17:**
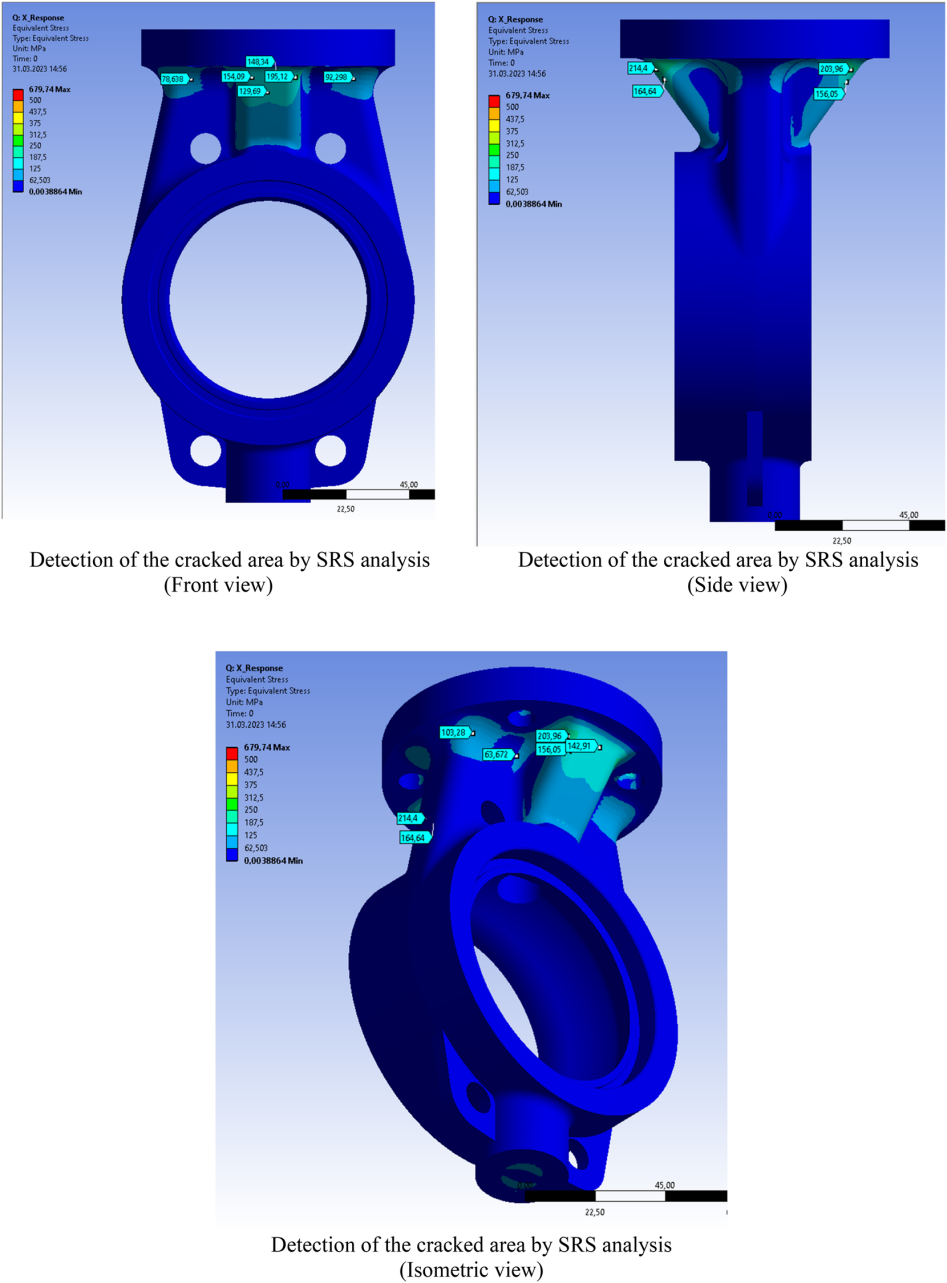
The new reinforced design SRS analysis (own design).

The ANSYS shock simulation for the new design was carried out with the same analyze parameters and no detection, red area, overstressed region was observed as seen in Fig. 17.

**Figure 18:**
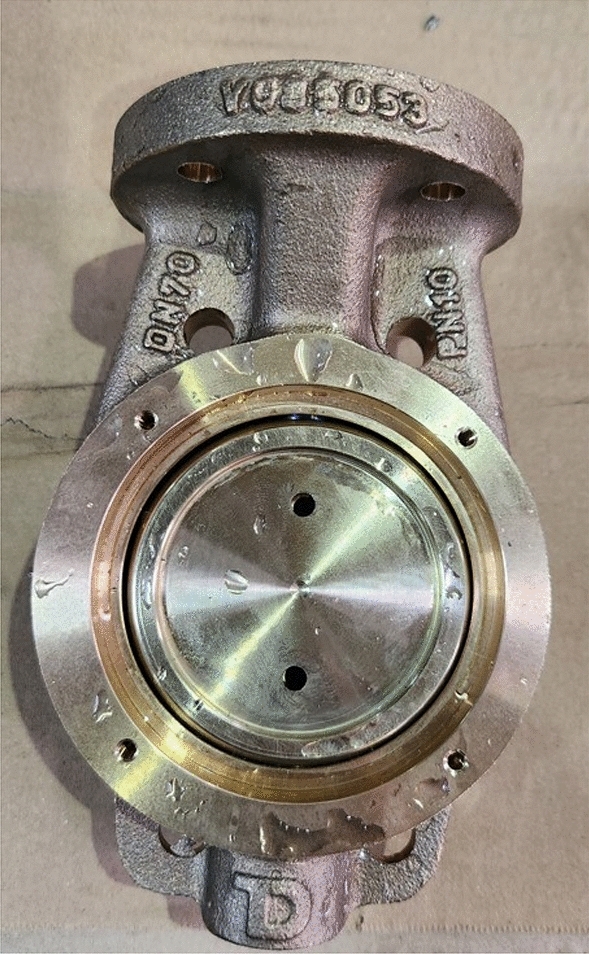
After the shock test image of the reinforced new design (own design).

The new design by reinforced sample was subjected to the shock test under the same conditions as the sample remaining from the previous test, and no mechanical and corrosion-induced damages were observed, including the same region, and passed the test successfully as detected in the ANSYS simulation. Figure 18 represents the image of the sample after the shock test.

## Results and discussion

The reinforced new design was passed without any detected area from the shock test. Comparing the cost of these improvements represents the engineering part of this study. The improvements made were discussed in terms of net weight, gross weight, and casting application speed. Both samples were made with CuSn10 material analysis, and the weight of the old design was 2.21 kg, and the weight of the new design was 2.64 kg. The effect of the new design on the gross weight after casting was determined to be only 430 g. As a 3-way feeder was used in the casting filling time, 4 s of saving was achieved, and casting was carried out in 15 s. The casting temperature was the same as 1150 °C. Moreover, fast, and low-cost production of the new design was planned by using old runner and feeder models. Comparison data between the test of the new reinforced design and the old design that failed the previous test are included in Table 3.

**Table 3:** The comparison of the old and reinforced new design with their parameters.

Parameters	Old design	Reinforced design
Shock Test	Failed	Passed
Material Type (C90700 / SAE 62, DIN 1075—2.1050, CC480K)	CuSn10	CuSn10
Production Process Temperature (°C)	1150	1150
Duration Time (Sec.)	19	15
Gross Weight (kg)	2.87	3.27
Net Weight (kg)	2.21	2.64
Feeders Type (#)	2	3

The reduced shock load and equivalent stress results in the x, y, and z directions with the new design are summarized in Table 4.

**Table 4:** Maximum stress and strain values of the shock simulation in the model.

Direction	Maximum equivalent stress [MPa]	Maximum equivalent strain [mm/mm]
X (Horizontal) Direction	136.37	0.0010
Y (Vertical) Direction	67.812	0.00044
Z (Horizontal) Direction	70.15	0.00045

The yield stress, the value at which plastic deformation starts, was defined as 130 MPa by considering the literature. On the other hand, the maximum rupture stress shall be as 270 MPa. These values were also defined in Table 2, too. By examining the data in Table 4, where the simulation results were described, it was determined that the structure provided sufficient strength under the shock profile loads in the x, y, and z directions of the structure under defined shock loads, and the stress results on the model remained below the limit stress value^38–41^.

Shock testing is generally performed to determine whether products can withstand high G force and short-term impact loads. This test is important in determining the level of fragility of products, it is especially critical to know how durable they will be during transportation and positioning of military materials. Materials show fracture damage in the form of brittle, ductile, creep and fatigue depending on their unloaded state, loading conditions and deformation. Ductile fracture involves a large amount of plastic deformation, and a significant amount of energy is released by the coalescence of micro-voids. Its crystallographic mode is slip and contains excessive plastic deformation^42–44^.

**Figure 19:**
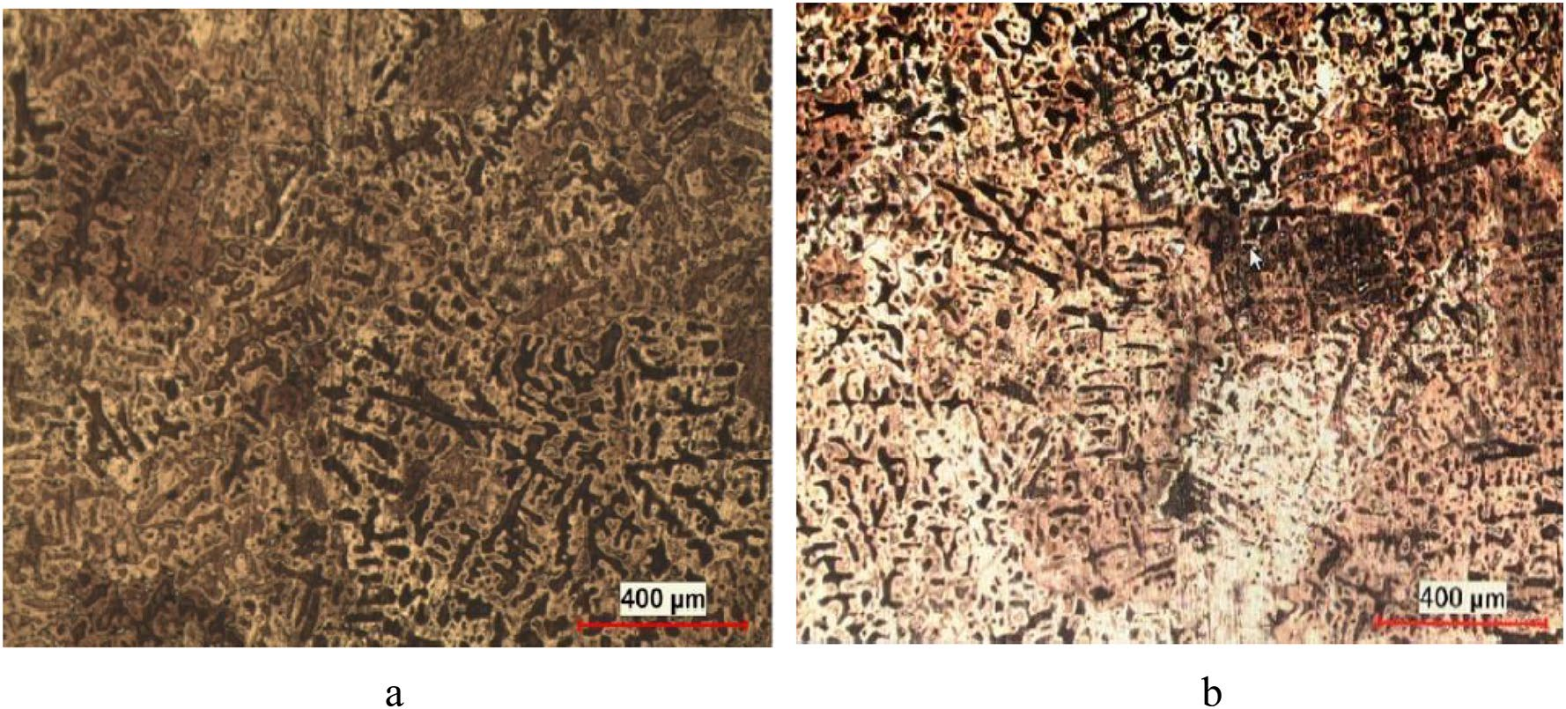
The microstructure of the designs (**a**) Failed from the shock test (**b**) Passed from the shock test.

Metallurgical characterization was carried out to support reinforcements that eliminate fracture in the shock test and microstructures were examined for this purpose. Figure 19 shows the microstructure comparison of the first and second designs. While no porosity was observed in both microstructures, the dendritic structure was observed more clearly in the sample produced with the reinforced design. This homogeneity resulted from the stable data of the improved system with the help of the casting simulation of the revised design. However, microstructural developments have been achieved by providing microstructural improvements as well as material thickening to prevent cracks.

**Figure 20:**
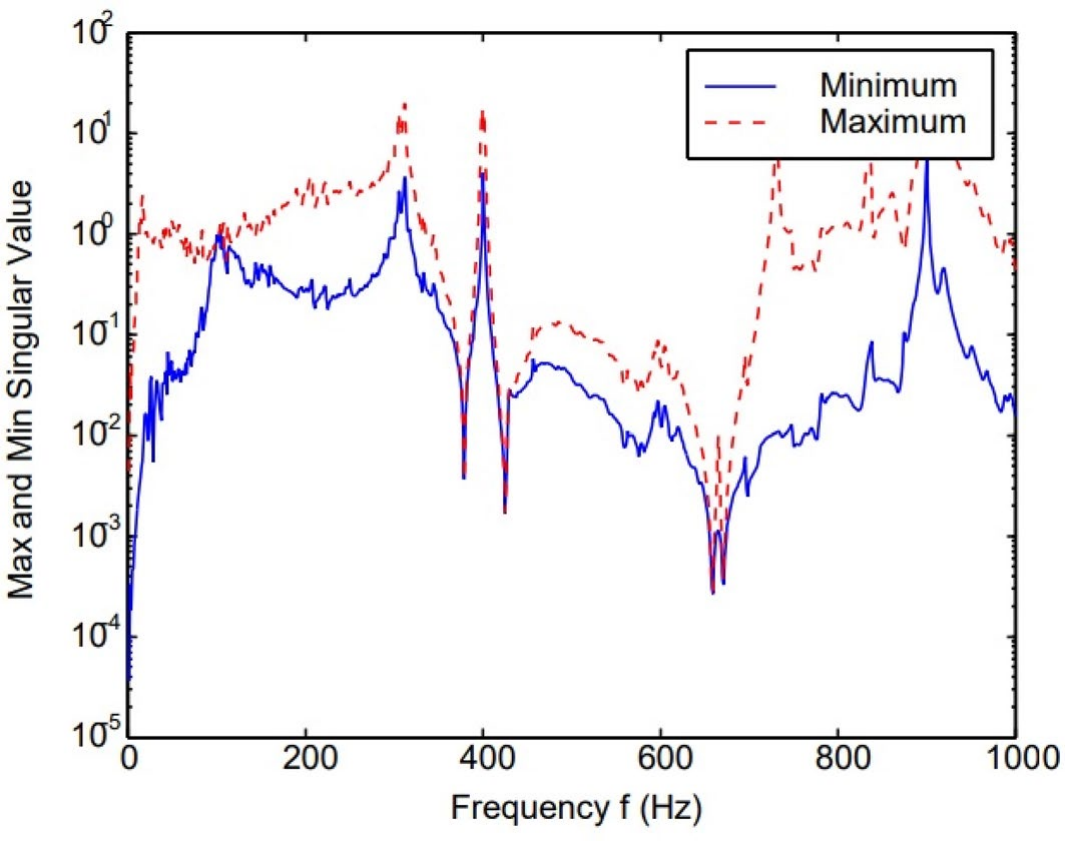
The error analysis during the experiment^45^.

Figure 20 indicates significant error from 0 to 400 Hz and small error from 420 to 700 Hz. The effect of structure impedance on error must be considered. A small error was amplified when stringer impedance was large. The proposed method offers the advantages of: (i) ease of application, (ii) error evaluation in the context of the master structure, and (iii) a means for considering all possible attachment-point velocities^45,46^.

It has been determined that the data related to the CAD of butterfly valves in mechanical shock testing for the naval defense industry have not been discussed in detail from the design to the final product in previous studies. In the literature, studies on CAD in shock tests can be found. Some of them include only design^47^, some include only testing^48^, and some include only applications^49^. In this study, all processes from design to final testing were explained in step-by-step detail.

Moreover, all the details from design to production, from characterization to mechanical shock tests were handled with data and this condition makes this study unique. Therefore, specific studies can be carried out for manufacturing sectors by considering the data given in this manuscript with details. Finally, with the digitalization of CAD in mechanical shock testing for defense industry, a significant increase in efficiency values on the casting was detected, and it is also quite effective in improving quality and reducing costs.

## Conclusions

In this study, CAD, simulation processes, prototype manufacturing and final product characterization processes of bronze butterfly valves, which are widely used in defense industry navy ships and submarines, were carried out. After the CAD processes, filling time–temperature, macro–micro shrinkage and particle tracking analyzes, and flow rates were determined for both designs with the simulation program. The selected material CuSn10 did not show any problems in terms of mechanical and corrosion properties. The resistance of the material exposed to the mechanical shock test is directly related to the design. The results obtained in the mechanical shock, stress, and deformation simulations of the analyzes made with the finite element method showed results equivalent to the real results. By revising the design with a safety factor of 18% on the specimen, it was ensured that the product could pass the mechanical shock test even at an acceleration of 4000 m/s^2^, then material become safe to use. With the 3-way feeding, the difference in net weight from 16% has been reduced to 12%, on the gross weight while the production time has been improved by 27%. This situation, which was seen as a disadvantage in net weight at first, was eliminated by reducing the gross weight and production time with the engineering approach and has become more advantageous. To support the results obtained, mechanical shock tests were carried out in the virtual environment with the finite element method in the ANSYS simulation environment, and tests were carried out in the real environment to verify the tests. There are numerous articles in the literature in which the effect of design on shock test resistance was investigated by handling them in a controlled manner, but what makes this study unique is that it was the first mechanical and metallurgically detailed investigation of the valves’ shock test method for the naval defense industry. All the stages were considered with details from the design to the simulation and then the real test values. According to this study, it was determined that especially with simulation supported designs, R&D prototype manufacturing and design verification, waste costs due to poor quality were reduced, and it was observed that the metallurgical properties of the parts produced with the runner design were directly affected. Another important output is that the results obtained with CAD in the simulation environment exactly match the prototype manufacturing results. In addition, this study paves the way for future research on not only bronze, but also steel, cast iron and even polymer products and serves as a guide. Finally, the simulation and CAD activities obtained during the design phase have critical importance in engineering applications, and in this study, CAD activities were discussed only in one phase. If it was handled in more phases, positive results could be achieved at once. For this reason, it is recommended that more CAD activities be addressed in future studies.

### Supplementary Information

Supplementary Information 1.

Supplementary Video 1.

Supplementary Video 2.

Supplementary Information 2.

Supplementary Information 3.

Supplementary Information 4.

## Data Availability

All data generated or analyzed during this study are included in this published article [and its supplementary information files].
