# Supplementary figures and images for: Mechanical shock test simulation analysis of butterfly valves developed for the naval defense industry and evaluation of real test and production data
Source: Sci Rep. 2024 Apr 27;14:9692. doi: 10.1038/s41598-024-60302-4 (PMC11055919; doi:10.1038/s41598-024-60302-4)

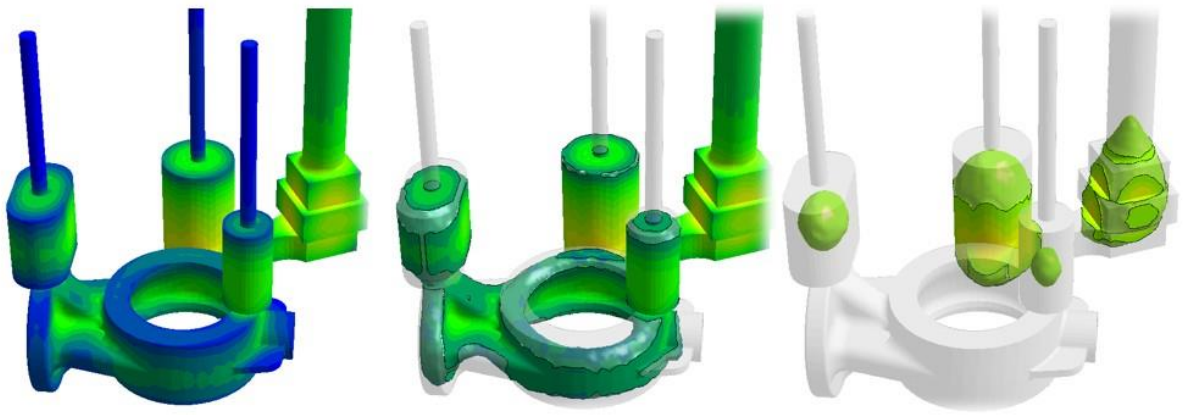

New design solidification simulation with three feeders

Supplement: Supplementary file 1 — Supplementary Information 1. [file 41598_2024_60302_MOESM1_ESM.pdf]
