# Supplementary material for: Mechanical shock test simulation analysis of butterfly valves developed for the naval defense industry and evaluation of real test and production data
Source: Sci Rep. 2024 Apr 27;14:9692. doi: 10.1038/s41598-024-60302-4 (PMC11055919; doi:10.1038/s41598-024-60302-4)
Supplement: Supplementary file 4 — Supplementary Information 2. [file 41598_2024_60302_MOESM4_ESM.pdf]

CLASS GUIDELINE

DNVGL-CG-0063 Edition April 2016

Shock testing of equipment and systems - Naval applications

The electronic pdf version of this document, available free of charge from http://www.dnvgl.com, is the officially binding version.

DNV GL AS

FOREWORD

DNV GL class guidelines contain methods, technical requirements, principles and acceptance criteria related to classed objects as referred to from the rules.

© DNV GL AS April 2016

Any comments may be sent by e-mail to *rules@dnvgl.com*

If any person suffers loss or damage which is proved to have been caused by any negligent act or omission of DNV GL, then DNV GL shall pay compensation to such person for his proved direct loss or damage. However, the compensation shall not exceed an amount equal to ten times the fee charged for the service in question, provided that the maximum compensation shall never exceed USD 2 million.

In this provision "DNV GL" shall mean DNV GL AS, its direct and indirect owners as well as all its affiliates, subsidiaries, directors, officers, employees, agents and any other acting on behalf of DNV GL.

CHANGES – CURRENT

This is a new document.

CONTENTS

Changes – current 3

Section 1 Introduction 5
- **1 Classification** 5

Section 2 Definitions 6
- **1 Contractual shock level** 6
- **2 Main functions** 6
- **3 Essential machinery and craft piping systems** 6
- **4 Other essential systems** 6
- **5 Important equipment** 6
- **6 Shock response spectra** 7

Section 3 Scope 8
- **1 General** 8

Section 4 Shock testing 9
- **1 General** 9
- **2 Recognised standards** 9
- **3 Shock levels** 9
- **4 Shock testing machines** 9
- **5 Test mounting** 10
- **6 Test procedure** 10
- **7 Meeting the required test severity** 11

Section 5 Certification process 13
- **1 General procedure** 13
- **2 Documentation** 13

Historic changes 15

SECTION 1 INTRODUCTION

1 Classification

When exposed to non-contact shock loads such as, but not limited to, underwater explosions from depth charges, naval ships run the risk of significant losses of operational effectiveness, mainly due to malfunctioning of systems caused by individual equipment failures.

This class guideline is intended to provide guidance on how to carry out relevant shock testing.

In addition, the user or owner may require defined operational functions to be verified to sustain defined shock levels. The functions may be broken down to a level so that it can be confirmed that all systems and equipment required for that function satisfy the requirement.

SECTION 2 DEFINITIONS

1 Contractual shock level

Shock design loads specified by the customer (yard or owner). The contractual shock level, as used in the system and component design, is represented by a shock response spectrum.

Normally one or two shock levels are specified based on the required shock protection of the different equipment on-board.

2 Main functions

The main functions are defined in RU SHIP Pt.1 Ch.1 Table 2 *Definitions.*

3 Essential machinery and craft piping systems

Machinery and piping systems in which failure will cause loss of one or more main functions, or cause deterioration of functional capability to an extent that the safety of the craft, personnel or environment is significantly reduced.

Typical essential systems are:

- cooling water systems (main engines)
- lubricating oil systems
- fuel oil systems
- feed-water, condensate and steam systems
- hydraulic oil systems
- compressed air systems
- ventilation systems
- exhaust systems
- bilge systems
- drainage systems
- ballast systems.

4 Other essential systems

These are systems in which failure will cause loss of one or more main functions, or cause deterioration of functional capability to an extent that the safety of the craft, personnel or environment is significantly reduced.

Other essential systems may be:

- power supply systems
- damage control systems
- fire-fighting system
- main or emergency switchboards and distribution boards or motor control centres for consumers serving essential systems or main functions
- emergency lighting system
- communication systems
- control and monitoring systems.

5 Important equipment

Equipment, which need not necessarily be in continuous operation for maintaining the craft's manoeuvrability, but which is necessary to maintain the crafts main functions.

Examples of important equipment are:

— fuel oil transfer pumps
— starting air compressors
— bilge and ballast pumps
— fire pumps
— ventilation fans for engine and boiler rooms
— side-thrusters
— converters, transformers and generators for supplying such equipment.

6 Shock response spectra

A shock response spectrum is the analytical visualisation of the combined forces and their duration within a defined frequency domain, acting on an assembly of mass-less oscillators.

SECTION 3 SCOPE

1 General

All essential machinery, craft piping systems, other essential systems, important equipment and control and monitoring equipment shall be designed and supported to resist the contractual shock level.

These systems and important equipment as defined above shall withstand the contractual shock level without:

— suffering serious degradation of functionality during and after the shock loading or
— experience permanent degradation of foundation and fastenings or
— have serious change of alignment.

The level of functionality may include acceptable damage, which shall be agreed upon by the involved parties, beforehand. This may incorporate:

— no degradation and fully functional; "shock grade 1"

  For all parts of the equipment which are necessary for the ship's safety and fulfilment of its combat task. Full function during and after shock load without reduction of performance. No loosening of parts which could endanger crew or other equipment of “shock grade 1”

— must remain safe; "shock grade 2".

All other parts of the equipment which are not essential for safety and fulfilment of combat task. No loosening of parts which could endanger crew or equipment of “shock grade 1”. Compliance with these requirements shall be documented by calculation or through shock testing. This class guideline deals with acceptable principles of shock testing in a laboratory environment, but does not deal with full-scale shock testing of an entire ship.

SECTION 4 SHOCK TESTING

1 General

It is important that the test method, the shock load and the response(s) are all clearly related to an acceptable standard and severity level before qualification tests are carried out.

2 Recognised standards

The following standards are typically used in shock testing:

i) Appendix C of ANSI S2.14-1973:
"*Methods for specifying the performance of shock machines".*

ii) Appendix B and Appendix C of IEC 68-2-27: 1987:
"Basic environmental testing procedures Part 2. Tests – Test EA and guidance: Shock".

iii) Annex B of ISO 8568- 1989(E):
"Mechanical shock – Testing Machines – characteristics and performance".

iv) STANAG 4549: NATO Standard:
"Testing of surface ship equipment on shock testing machines".

Note:

Many of the technical reference in this class guideline are based on an approach described in STANAG 4549.

Other standards, like MIL-S-901, BV 0430, BV 0230 / D5050-0599 may upon consideration of applicability be used.

---e-n-d---o-f---n-o-t-e---

3 Shock levels

Typically a range of shock levels may be defined by idealised shock response spectra. A number of navies have different design philosophies, which may take into account location of equipment on board (i.e. shock zones) or other parameters.

Testing is normally to be based on a defined shock level specified in terms of a shock response spectrum.

One acceptable definition of shock level is defined in STANAG 4549. In this standard, the required shock response spectrum level is defined by relative displacement, pseudo velocity and absolute acceleration within certain frequency bands, and are shown as three intersecting straight lines in a log-amplitude vs log frequency diagram. Such a shock response spectrum is denoted as NS ([Relative displacement – mm]; [Pseudo velocity – m/s]; [absolute acceleration – m/s^2]).

Example:

NS (0.035; 3.5; 1250) means a shock response spectrum with:

— 35 mm relative displacement between 4.0 Hz and 15.9 Hz
— 3.5 m/s pseudo velocity between 15.9 Hz and 56.8 Hz and
— ~125 g (1250 m/s^2) absolute acceleration between 56.8 Hz and 400 Hz.

Other approaches than defined in STANAG 4549 may be accepted upon consideration.

Acceptable approaches may include but not be limited to:

— a time domain approach
— a charge of X kg explosive of type Q at a distance of Y meters and a depth of Z meters.

4 Shock testing machines

Shock testing machines must be able to produce the required shock level. The agency carrying out the tests must document the accuracy and capabilities of test machines.

It is not required to use a shock-testing machine, which has been certified for such application. However, where such testing machine is not used, well defined shock loads measurements must be made prior to the acceptance tests at locations close to the attachment points of the equipment. The measurements must show that the corresponding shock level is equal to or exceeds the level specified for the test.

5 Test mounting

Test of equipment shall also include the mounting arrangement with which the equipment is attached to its foundation. Where such mountings are not part of the equipment delivery, the mounting arrangement shall be simulated during testing.

When such an arrangement, i.e. the equipment on mountings, has completed the shock testing, the equipment without mountings or on another type of mounting shall not be considered certified without separate testing.

The test arrangement should simulate as closely as possible the final intended application. If cabling or piping represent a significant effect on stiffening, this should also be simulated during the shock testing.

Wherever possible the equipment has to be in operation mode during the test and the functions have to be checked during and after the test.

If, because of limitations of the test facility, it is not possible to test resiliently mounted equipment together with the mountings, these shall be tested on fixed support using the residual shock above the mounting as derived by calculations.

If it is not possible to test the full equipment assembly, as a minimum, the most critical components, as agreed between the navy and the Society, shall be tested.

6 Test procedure

6.1 Shock directions and attachment to the shock testing machines

Shock in three different shipboard directions must be simulated by three separate tests.

In decreasing severity;

a) vertically (sense upwards)
b) athwart-ships (sense not specified)
c) fore and aft direction (sense not specified).

Often the orientation of the equipment on board is not known at the time the equipment is tested. By defining, for a piece of equipment, 3 specific perpendicular equipment axes X, Y and Z it is possible to distinguish 3 different cases as shown in Table 1.

Table 1 Tests in the equipment directions X, Y, and Z

	Case 1	*Case 2*	*Case 3*
X	(a)	(a)	(a)
Y	(b)	(b)	(a)
Z	(c)	(b)	(a)

Case 1 Applies if there is only a single orientation on board possible, which is fully known.

Case 2 Applies if it is only clear that the topside of the equipment points upwards (here the X-axis) in all cases. This is the most common case, in particular for smaller equipment.

Case 3 Applies to a few pieces of equipment, which can be mounted in any orientation.

Case 2 and in particular case 3 require more severe overall testing than case 1.

7 Meeting the required test severity

7.1 Test severity

Typical test severity will be defined for the vertical direction (see STANAG 4549):

i) For the test in the athwart-ships direction a similar spectrum should be used having as a minimum 50% values for the vertical test.
ii) For the test in the fore and aft direction a similar spectrum should be used having as a minimum 25% of the values for the vertical test.

There are always three of these maximum severity tests in the X–, Y– and Z– direction of the equipment, but it depends on the particular case (1, 2 or 3) above, as to which of these tests as mentioned under (a), (b) and (c) are carried out.

A test series should comprise 3 successive shocks in each direction as a minimum.

Additional maximum severity tests may be required if different modes or conditions of operation shall be represented during the tests.

7.2 Shock motion measurements

The shock, the equipment is subjected to must be measured, taking into account the following requirements:

— The direction of the transducers must be parallel to the equipment axis (X, Y or Z) for which the actual test is being carried out.
— The transducers must be installed to measure the shock input as close as possible to the points where the equipment is attached to the shock testing machine (or fixtures of the machine). Or as close as possible to a mounting, on that side of the mounting that is connected to the shock testing machine (or fixtures of the machine). The maximum distance must not be more than 50 mm. The stiffest location possible must be chosen.
— If all attachment points (mountings) are in a single plane, two transducers must be installed diametrically with respect to the equipment.
— If attachment points are in two planes, for instance a cabinet mounted both to a deck and a bulkhead, three transducers must be installed. Two of them diametrically in that plane which takes most of the shock load, The third one in the other plane at a location to be selected far away from the other transducers.

7.3 Comparison of spectra

The required shock level is considered to have been met during testing if all measured spectra exceed the required spectra, with the exception of minor excursions as defined in the selected test standard.

Only the three maximum severity tests need to be considered. See Table 1.

The requirement must be met for each of the 2 or 3 measurement locations individually and per shock.

Averaging of measured spectra is not allowed.

It is not permitted to use envelopes of shock response spectra for the same measuring location as obtained during successive shocks.

Note:

A shock response spectrum can be transferred to a pulse with a certain shape, amplitude and duration. Given a certain pulse, comparison can be made in the time domain. Such alternative test methods are acceptable to the Society upon consideration.

---e-n-d---o-f---n-o-t-e---

7.4 Tolerances

Tolerances shall be in accordance with the shock test standard used.

7.5 Required functioning

It may be feasible that some damage and or reduced functioning of equipment during and following shock testing is acceptable to the purchasing authority and class.

Besides indicating all required operational conditions for the tested equipment, the procurement document must clearly state the applicable acceptance criteria and functional requirements for each mode of operation tested. In other words, for each performance criterion specified in the procurement document, a corresponding shock test acceptance criterion shall be developed which indicates the degree, if any, of degradation of performance allowable as a result of exposure to shock.

Where such a definition has not been agreed upon before testing commences, the functional requirements following the test shall be the same as those prior to the test.

SECTION 5 CERTIFICATION PROCESS

1 General procedure

Typical certification will normally involve the following parties:

Navy (owner)	The navy will specify the required shock level with reference to a recognised standard. Any acceptable reduction in functionality following shock should also be specified.
Shipyard	The shipyard will order equipment from a manufacturer with these specified shock requirements. Shipyard will maintain all certificates for purchased equipment for review by class representative at the shipyard. Information on orientation of equipment, required direction of testing and required functionality after shock testing should be agreed with navy and class. This information should be a part of the specification to the equipment manufacturer.
Manufacturer	The equipment manufacturer will design and manufacture equipment according to the specification. The manufacturer will normally issue a data book for his equipment accompanied by relevant certificates, including the shock test certificate.
Test laboratory	The test agency or laboratory will carry out shock testing of the equipment. Based on results of testing the test agency or laboratory will normally issue a test report documenting test procedure and test results.
Class	Class will verify shock testing at test laboratory and issue to the test laboratory a certificate of compliance. Where equipment is also subject to certification as a result of other class requirements than shock testing, class will also carry out relevant certification at the manufacturer.

Communication between the various parties will be necessary in order to ensure that the delivered product meets the project specifications.

Note that the certificate will document a certain shock resistance. However, suitability of the equipment for a specific application should also consider the following:

a) If the equipment is on mountings during the tests, the same type mountings shall be used in the final application. The mounting arrangement and relevant effects of any other attached equipment will need to be informed to the test agency or laboratory either by the manufacturer or the shipyard.
b) For the intended orientation on board, the relevant tests will have to be satisfied.
c) Acceptability of any possible damage, malfunction or degradation of performance as noted during testing must be agreed upon.
d) The equipment being offered is of identical design and fabrication to that which is being tested.

A manufacturer or shipyard may in certain circumstances not have a specific application determined before delivery. In such cases a standard shock level and testing in varying positions and directions may be specified. Suitability for an application will then have to be determined when final project information is available.

2 Documentation

2.1 Test report

In order to document results of the shock testing, a test report from a test laboratory shall be made. This report should include the following:

Equipment description

— manufacturer data
— unique identification of equipment to be tested
— description of actual equipment as tested

— weight and overall dimensions
— description of any mounting arrangement used to support the equipment.

Description of shock testing

— project reference and required shock level
— reference to recognised standard
— description of shock testing equipment
— certification or equivalent of shock testing equipment
— description of how equipment was mounted in the test machine
— location of measuring devices
— certification and calibration of measuring devices
— table showing the sequential numbered tests, test directions, test severity
— table showing shock response results.

Damage and performance tests

— definition of agreed acceptable degradation of functionality if agreed on previous to shock testing
— performance tests on equipment or any of its components, carried out during, or at the end of the tests shall be described including all functions controlled and any changes in performance observed
— any damage that has occurred as a result of a shock test shall be noted.

Conclusions and comments

— test agency or laboratory’s conclusion regarding test results and acceptance criteria
— verified shock level with respect to recognised standard
— any comments deemed relevant.

2.2 DNV GL certificate

DNV GL will verify results of the shock testing. The verification will normally comprise witness of the actual testing and review of the laboratory test report.

A statement of compliance will be issued.

The statement will contain the following information:

— manufacturer data
— description and identification of the equipment tested
— project reference
— reference to test method
— reference to test report
— shock rating achieved
— date and signature of the acceptance authority
— any comments on:
    — functional testing
    — mounting arrangement
    — test position/direction.

Note:

Certification of shock properties may be included in the DNV GL type approval scheme.

---e-n-d---o-f---n-o-t-e---

HISTORIC CHANGES

There are currently no historical changes for this document.

DNV GL

Driven by our purpose of safeguarding life, property and the environment, DNV GL enables organizations to advance the safety and sustainability of their business. We provide classification and technical assurance along with software and independent expert advisory services to the maritime, oil and gas, and energy industries. We also provide certification services to customers across a wide range of industries. Operating in more than 100 countries, our 16 000 professionals are dedicated to helping our customers make the world safer, smarter and greener.

SAFER, SMARTER, GREENER
