# Supplementary material for: Mechanical shock test simulation analysis of butterfly valves developed for the naval defense industry and evaluation of real test and production data
Source: Sci Rep. 2024 Apr 27;14:9692. doi: 10.1038/s41598-024-60302-4 (PMC11055919; doi:10.1038/s41598-024-60302-4)
Supplement: Supplementary file 6 — Supplementary Information 4. [file 41598_2024_60302_MOESM6_ESM.pdf]

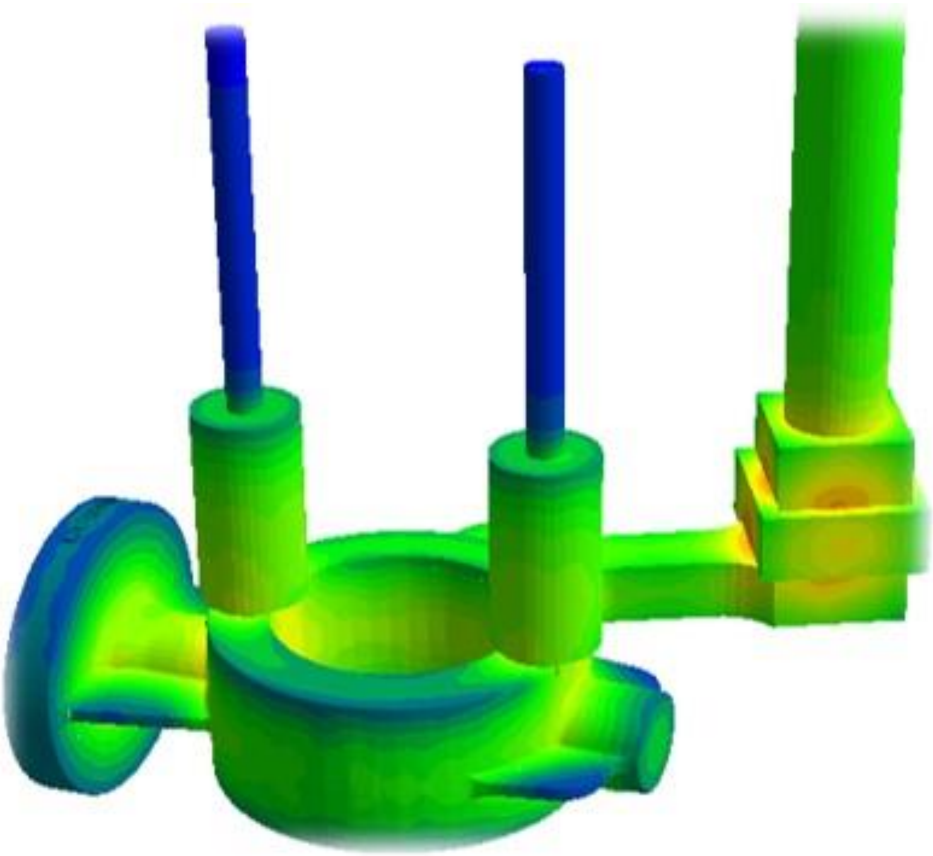

The solidification simulation of the sample remaining from the shock test during casting was carried out and the design was revised accordingly.
